# The impact of instagram addiction on self esteem in Tunisian doctors

**DOI:** 10.1192/j.eurpsy.2023.1409

**Published:** 2023-07-19

**Authors:** N. Messedi, S. Kolsi, E. Fakhfakh, I. Chaari, F. Charfeddine, L. Aribi, J. Aloulou

**Affiliations:** Psychiatry « B » Department, Hedi Chaker University Hospital Center, Sfax, Tunisia

## Abstract

**Introduction:**

Instagram, one of the most widely used social media by population was changed the way individuals communicate around the world. It can be used to increase individual self popularity or increase self-esteem.

**Objectives:**

To determine the relationship between the instagram addiction and self-esteem in a population of tunisian doctors.

**Methods:**

A cross-sectional study was conducted online using the Google Forms platform, with a sample of tunisian doctors in september and october 2022. We used :

- Instagram Addiction Scale (IAS) : to assess Instagram addiction levels. A score above 37 indicates addiction to instagram.
- Rosenberg Self-Esteem Scale : to measure the level of self-esteem. Higher scores indicate higher levels of self-esteem.

**Results:**

The sample comprised 106 doctors.

- IAS : The mean score was 31.18 (SD=11.64). Less than half (42.5%) presented an addiction to instagram and 36.8% were mildly addicted.
- The mean self-esteem score was 29.70 (SD=3.57). Less than half (44%) had low to very low self-esteem.
- The instagram addiction score was negatively correlated with the self-esteem score (r=-0.543 ; p=0.0001).

It was found that these scores were significantly lower in the addicts (p=0.0001) indicating lower self-esteem. Among addicts, 80% had low to very low self-esteem. Among non addicts, 18% had low to very low self-esteem.

**Conclusions:**

It was found that the higher the intensity of using instagram the lower the self esteem.

Further research is expected to be carried out with a wider population to verify the present findings and to study other factors related to instagram addiction such as loneliness and life satisfaction.

**Disclosure of Interest:**

None Declared

